# Supplementary material for: Fabrication of Noble-Metal-Free Mo2C/CdIn2S4 Heterojunction Composites with Elevated Carrier Separation for Photocatalytic Hydrogen Production
Source: Molecules. 2023 Mar 9;28(6):2508. doi: 10.3390/molecules28062508 (PMC10057527; doi:10.3390/molecules28062508)
Supplement: Supplementary file 1 [file molecules-28-02508-s001.zip › molecules-2189652-supplementary.pdf]

Fabrication of Noble-Metal-Free $Mo_2C/CdIn_2S_4$ Heterojunction Composites With Elevated Carrier Separation for Photocatalytic Hydrogen Production

Hong Qiu, Xiaohui Ma, Hongxia Fan, Yueyan Fan, Yajie Li, Hualei Zhou* and Wenjun Li*

Beijing Key Laboratory for Science and Application of Functional Molecular and Crystalline Materials, University of Science and Technology Beijing, Beijing 100083, China

*Correspondence: wjli_ustb@163.com, hlzhou@ustb.edu.cn

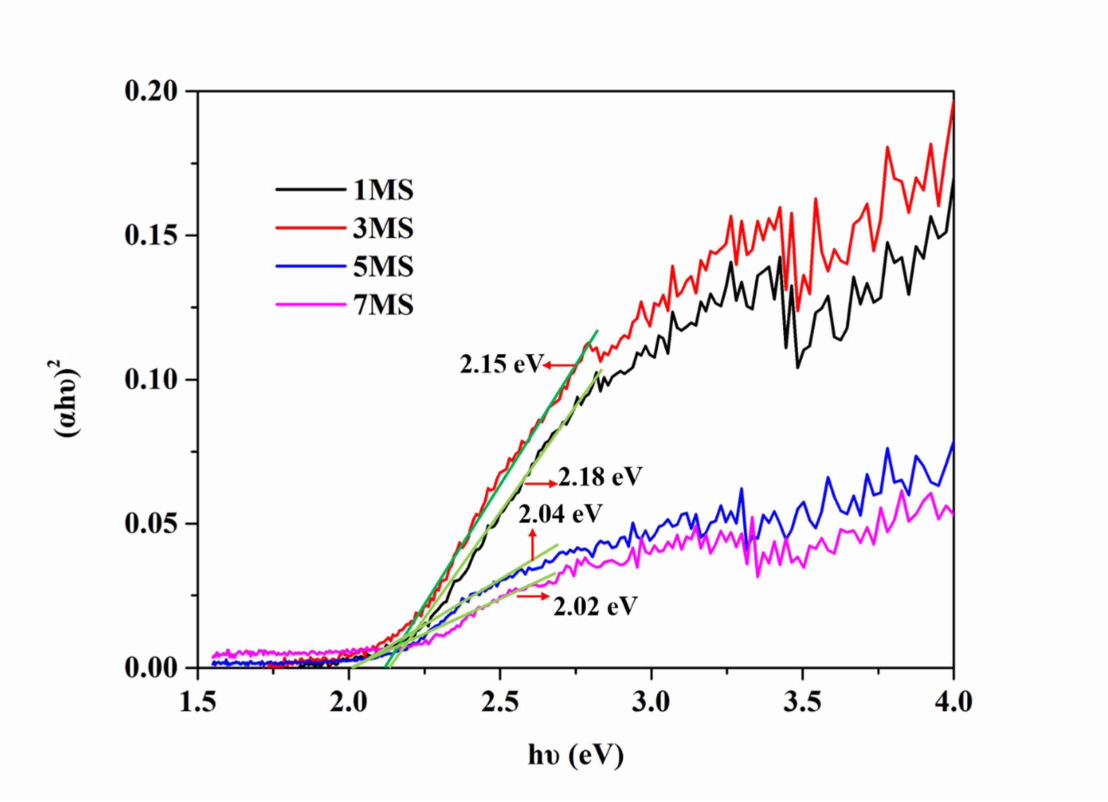

Fig. S1 Tacu's curve of MS heterojunction composites.

Table S1. The incident radiation intensity entering the photoreactor is shown in the table below.

	1	2	3	4	5	6	7	8	9
Optical power density (mW/cm^2)	146.5	45.4	56.7	63.6	40.6	53.3	34.8	53.7	50.4

Average value (mW/cm^2)	60.6
Reactor area (cm^2)	19.6
Incident radiation intensity (mW)	1186.9

We measured the intensity of the incident radiation entering the photoreactor by an optical power meter (CEL-2000-2, China). Calculate the intensity of incident radiation by measuring the optical power density at ten different positions of the photoreactor according to the following formula:

$$E=P_{Average}*A$$

Where E, $P_{Average}$ and A stand for the intensity of the incident radiation, the power density of light and the irradiation area, respectively.

Table S2. Comparison of hydrogen evolution data of $Mo_2C/CdIn_2S_4$ composites compared with other literature reports.

Photocatalysts	Noble metal	H_2 evolution	Ref.
$Mo_2C/CdIn_2S_4$	No	1178.32 μmol g^{-1} h^{-1}	This work
$MoP/CdIn_2S_4$	No	286.10 μmol g^{-1} h^{-1}	[1]
$Co_2P/CdIn_2S_4$	No	471.87 μmol g^{-1} h^{-1}	[2]
$Co_9S_8/CdIn_2S_4$	No	1083.6 μmol g^{-1} h^{-1}	[3]
$CdIn_2S_4$/CNFs/Co_4S_3	No	25.87 mmol g^{-1} h^{-1}	[4]
$ZnIn_2S_4/CdIn_2S_4$	0.75 wt% PdS	780 μmol h^{-1}	[5]
$MoS_2/CdIn_2S_4$	No	1868.19 μmol g^{-1} h^{-1}	[6]
$MoS_x/CdIn_2S_4$	No	2846.73 μmol g^{-1} h^{-1}	[7]
$In_2S_3/CdIn_2S_4/In_2O_3$	Pt	2004 μmol g^{-1} h^{-1}	[8]

References

[1] X. Ma, W. Li, C. Ren, M. Dong, L. Geng, H. Fan, Y. Li, H. Qiu, T. Wang, Construction of novel noble-metal-free $MoP/CdIn_2S_4$ heterojunction photocatalysts:

Effective carrier separation, accelerating dynamically H_2 release and increased active sites for enhanced photocatalytic H_2 evolution, J Colloid Interface Sci, 628 (2022) 368-377.
[2] X. Ma, W. Li, H. Li, M. Dong, L. Geng, T. Wang, H. Zhou, Y. Li, M. Li, Novel noble-metal-free $Co_2P/CdIn_2S_4$ heterojunction photocatalysts for elevated photocatalytic H_2 production: Light absorption, charge separation and active site, Journal of Colloid and Interface Science, 639 (2023) 87-95.
[3] C. Li, Y. Zhao, X. Liu, P. Huo, Y. Yan, L. Wang, G. Liao, C. Liu, Interface engineering of $Co_9S_8/CdIn_2S_4$ ohmic junction for efficient photocatalytic H_2 evolution under visible light, J Colloid Interface Sci, 600 (2021) 794-803.
[4] S. Guo, Y. Li, C. Xue, Y. Sun, C. Wu, G. Shao, P. Zhang, Controllable construction of hierarchically $CdIn_2S_4/CNFs/Co_4S_3$ nanofiber networks towards photocatalytic hydrogen evolution, Chemical Engineering Journal, 419 (2021) 129213.
[5] Y. Yu, G. Chen, G. Wang, Z. Lv, Visible-light-driven $ZnIn_2S_4/CdIn_2S_4$ composite photocatalyst with enhanced performance for photocatalytic H_2 evolution, International Journal of Hydrogen Energy, 38 (2013) 1278-1285.
[6] B. Zhang, H.X. Shi, X.Y. Hu, Y.S. Wang, E.Z. Liu, J. Fan, A novel S-scheme $MoS_2/CdIn_2S_4$ flower-like heterojunctions with enhanced photocatalytic degradation and H_2 evolution activity, Journal of Physics D: Applied Physics, 53 (2020) 205101.
[7] Q. Li, W.L. Liu, X.J. Xie, X.L. Yang, X.F. Chen, X.G. Xu, Synthesis and Characterization of Amorphous Molybdenum Sulfide $(MoS_x)/CdIn_2S_4$ Composite Photocatalyst: Co-Catalyst Using in the Hydrogen Evolution Reaction, Catalysts 2020, 10, 1455.
[8] D. Ma, J.W. Shi, Y. Zou, Z. Fan, J. Shi, L. Cheng, D. Sun, Z. Wang, C. Niu, Multiple carrier-transfer pathways in a flower-like $In_2S_3/CdIn_2S_4/In_2O_3$ ternary heterostructure for enhanced photocatalytic hydrogen production, Nanoscale, 10 (2018) 7860-7870
